# Integrating deep learning and clinical characteristics for early prediction of endometrial cancer using multimodal ultrasound imaging: a multicenter study

**DOI:** 10.3389/fonc.2025.1600242

**Published:** 2025-07-08

**Authors:** Cuiyan Lin, Wanming Chen, Jichuang Lai, Jieyi Huang, Xiaolu Ye, Sijia Chen, Xinmin Guo, Yichun Yang

**Affiliations:** ^1^ Department of Ultrasound, Guangzhou Red Cross Hospital, Guangzhou, Guangdong, China; ^2^ Department of Ultrasound, The First Clinical Medical College of Guangzhou University of Chinese Medicine, Guangzhou, Guangdong, China; ^3^ The First Clinical Medical College of Guangzhou University of Chinese Medicine, Guangzhou, China

**Keywords:** endometrial cancer, predictive model, ultrasound imaging, clinical risk factors, deep learning

## Abstract

**Background:**

Endometrial cancer (EC) is one of the most prevalent malignancies affecting the female reproductive system. It poses significant health risks to women and imposes a substantial economic burden on healthcare systems. Early and accurate diagnosis is critical for improving patient outcomes. While traditional diagnostic methods rely on clinical evaluation and imaging, there is growing interest in leveraging artificial intelligence, particularly deep learning (DL), to enhance diagnostic accuracy.

**Methods:**

This study developed a DL-based predictive model integrating multimodal ultrasound features and clinical risk factors to improve early EC diagnosis. A retrospective, multicenter analysis was conducted using 1,443 multimodal ultrasound images—including two-dimensional (2D) and color Doppler images—from 611 patients, of whom 132 were diagnosed with EC and 479 were non-EC cases. Clinical risk factors such as body mass index (BMI), menopausal status, irregular vaginal bleeding, and hypertension were identified as significant predictors (P < 0.05) and incorporated into a clinical model. Separate DL models were trained on 2D and color Doppler ultrasound images, and their performance was evaluated individually and in combination with the clinical model.

**Results:**

The area under the receiver operating characteristic curve (AUC) for the clinical model was 0.772 (95% CI: 0.690–0.854). The 2D and color Doppler DL models achieved AUCs of 0.792 (95% CI: 0.719–0.864) and 0.813 (95% CI: 0.745–0.881), respectively. When combined with the clinical model, the merged model demonstrated superior predictive performance. In the external validation cohort, the merged model achieved an AUC of 0.892 (95% CI: 0.846–0.938), indicating high diagnostic accuracy.

**Conclusions:**

The integration of multimodal ultrasound imaging and clinical risk factors using DL significantly enhances the accuracy of endometrial cancer diagnosis. The merged model demonstrated strong generalizability in external validation, underscoring its potential clinical utility. Future studies should focus on larger, prospective multicenter trials to further validate these findings and explore the implementation of this approach in personalized patient care.

## Introduction

Endometrial cancer (EC) is the sixth most common cancer among women, with an estimated 420,242 new cases diagnosed globally in 2022 (1). The incidence of EC is increasing annually, with approximately 142,000 new cases reported each year worldwide (2), and a growing trend toward younger onset. EC often develops insidiously, and by the time clinical symptoms manifest, the disease has often progressed to an advanced stage, leading to poorer prognoses. In particular, the serous subtype of EC accounts for nearly 40% of EC-related deaths, highlighting the urgent need for improved early detection strategies (3).

Transvaginal ultrasound (TVUS) is widely used as a first-line screening method due to its non-invasive, real-time, rapid, and cost-effective nature. In postmenopausal women, an endometrial thickness threshold of 5 mm has been shown to provide high sensitivity for EC detection. However, its specificity remains low at 51.5%, necessitating additional diagnostic procedures for most women to confirm or rule out EC (4, 5). Furthermore, advanced modalities such as three-dimensional (3D) ultrasound, often utilizing 3D Doppler indices, have also become integral to routine gynecological practice (6, 7). In premenopausal women, physiological fluctuations in endometrial thickness further reduce specificity, leading to diagnostic challenges. Alternative diagnostic methods, such as hysteroscopy, are often limited by their invasive nature, associated surgical risks, and can cause significant discomfort or severe pain, which may also be accompanied by challenges in obtaining adequate or representative tissue samples. While magnetic resonance imaging (MRI) is effective for preoperative assessment, it is neither cost-effective nor practical for routine EC screening. Computed tomography (CT) is primarily used for detecting metastases in the chest, abdomen, and pelvis but is associated with radiation exposure, making it unsuitable for screening purposes (8). These limitations underscore the urgent need for a novel, non-invasive screening method that allows for accurate early detection of EC (9).

Recent advances in artificial intelligence (AI), particularly deep learning (DL) applications in medical imaging, offer promising opportunities to enhance ultrasound-based diagnostics. DL algorithms, particularly convolutional neural networks (CNNs), leverage multi-layered artificial neural networks to automatically extract and learn hierarchical imaging features from large datasets. These algorithms excel at detecting subtle morphological and vascular patterns in tumor imaging, enabling precise lesion characterization. Previous studies have demonstrated that DL-enhanced ultrasound imaging can outperform conventional diagnostic approaches (10). However, the integration of AI-driven imaging features with clinical risk factors remains underexplored (11–15).

This study aims to address this gap by employing a multimodal, multicenter, retrospective design to integrate AI-driven ultrasound imaging with clinical indicators. By leveraging DL architectures, we seek to enhance early detection and risk stratification in EC, ultimately contributing to improved clinical outcomes.

## Methods

### Study design

This multicenter, retrospective study was conducted between 2022 and 2024 at two research centers: Center 1 (The First Affiliated Hospital of Guangzhou University of Chinese Medicine) and Center 2 (Guangzhou Red Cross Hospital). Center 1 contributed a total of 351 patients, including 81 EC cases and 270 non-EC cases, which were used as the training set. Center 2 provided a total of 260 patients for external validation, comprising 51 EC cases and 209 non-EC cases. Multimodal ultrasound images—including two-dimensional and color Doppler images—as well as clinical data were collected from patients with pathologically confirmed EC. This study was conducted in accordance with the Declaration of Helsinki, and approved by the institutional review board (IRB). The requirement for informed consent was waived due to the retrospective nature.

**Figure 1:**
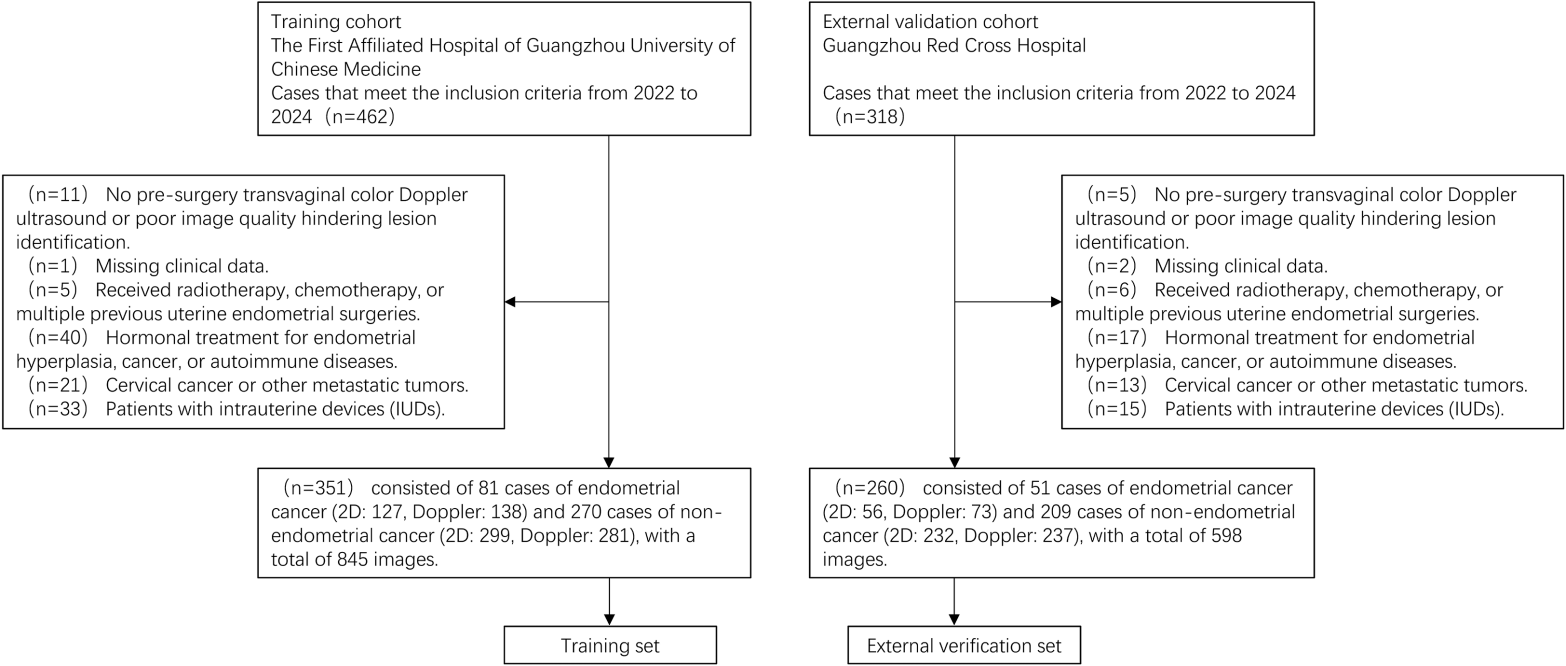
The flowchart of study population selection.

The inclusion criteria are as follows: (1) Patients who underwent endometrial aspiration biopsy, curettage, or hysterectomy with pathologically confirmed diagnoses between 2022 and 2024. (2) Preoperative transvaginal color Doppler ultrasound performed according to standardized protocols. Exclusion criteria are as follows: (1) Poor-quality ultrasound images or absence of preoperative transvaginal color Doppler ultrasound. (2) Incomplete clinical data. (3) History of prior radiotherapy, chemotherapy, or multiple endometrial surgeries. (4) Use of hormone therapy for endometrial hyperplasia or autoimmune diseases. (5) Diagnosis of cervical cancer or other malignancies. (6) Presence of intrauterine devices obstructing endometrial visualization. **Figure 1** presents the flowchart of the study population selection.

In the training set, 81 EC cases were included, with 127 two-dimensional images and 138 color Doppler images, while 270 non-EC cases contributed 299 two-dimensional images and 281 color Doppler images, resulting in a total of 845 images. For the external validation set, 51 EC cases were included, with 56 two-dimensional images and 73 color Doppler images, while 209 non-EC cases contributed 232 two-dimensional images and 237 color Doppler images, amounting to 598 images. Overall, the dataset comprised a total of 1,443 ultrasound images.

### Acquisition of ultrasound images

Transvaginal ultrasound (TVU) examinations were performed by three experienced physicians using various scanner models, including GE Voluson E6, GE Voluson E8, GE Voluson E10, Hitachi Avius L, Philips EPIQ 5, and Toshiba Aplio500. All systems were equipped with high-frequency (5–14 MHz) transvaginal probes. The examining physicians possessed extensive experience (>15 years) in obstetric and gynecologic ultrasound and strictly adhered to standardized examination and measurement techniques as outlined in the IETA consensus statement (16). Standard two-dimensional TVU and color Doppler ultrasound images, specifically showing the uterine endometrium and any endometrial lesions, were acquired.

### Model development

In this study, we developed DL models based on two imaging modalities: two-dimensional (2D) ultrasound and color doppler ultrasound. The training set included Research Center 1 (n=351; images=845), while Research Center 2 (n=260; images=598) was designated as the external validation set. We selected four distinct and well-established convolutional neural network (CNN) architectures: ResNet-50 (17), ResNet-152 (18), EfficientNet-B0 (19), and DenseNet-201 (20). These architectures are known for their strong performance on natural image classification tasks. To expedite the training process, we employed transfer learning by freezing the pre-trained convolutional layers and only training the fully connected layers. Each architecture was trained separately on both imaging modalities. The model demonstrating the best performance, as validated by an external validation set, was then selected as the final DL model.

Before training, preprocessing techniques such as image normalization, resizing (to 512×512 pixels), and data augmentation (including random vertical and horizontal flipping, rotation, grayscale transformation, and adjustments to brightness, contrast, saturation, and hue) were applied to reduce overfitting and enhance training performance. Each model was trained using five-fold cross-validation, and hyperparameters such as batch size (16), learning rate (3e-5), and the number of epochs (200) were optimized. The Adam optimizer, with β1 of 0.9, β2 of 0.999, epsilon of 1e-8, and weight decay set to 0.01 for L2 regularization, was employed to prevent overfitting. Considering the EC-to-non-EC ratio is significantly imbalanced, we employed a class-weighted cross-entropy loss function. Specifically, a higher weight was assigned to the EC class. By increasing the cost of misclassifying an EC case, the loss function effectively forces the model to pay more attention to these rare but critical instances, thus improving its ability to correctly identify. After training, the model’s performance was evaluated on the external validation set using key metrics such as the area under the receiver operating characteristic curve (AUC), accuracy, sensitivity, specificity, and F1 score. The loss and accuracy curves for both imaging modalities are presented in **Supplementary Figure S1**.

### Image-level to patient-level conversion

Since clinical diagnoses are typically made at the patient level, some patients in this study had multiple ultrasound images (ranging from 1 to 8 per patient), which could introduce bias. To mitigate this, we averaged the image-level risk scores to generate a patient-level score, thereby reducing potential bias associated with multiple images per patient.

### Clinical risk factor screening and model development

In addition to imaging data, we collected baseline clinical variables, including age, body mass index (BMI), history of gestation, menopausal status, presence of irregular vaginal bleeding, and history of hypertension, diabetes, hypothyroidism, and polycystic ovary syndrome (PCOS). Univariate logistic regression was performed to identify statistically significant clinical predictors (P < 0.05), which were then subjected to multivariate regression analysis. Variables that remained significant in the multivariate analysis were incorporated into the final clinical model for EC prediction.

### Combined model

A combined model was developed by integrating the best-performing CNNs models from the two-dimensional ultrasound and color Doppler modalities with the clinical prediction model. This hybrid approach aimed to enhance the accuracy and reliability of EC prediction.

### Statistical Analysis

Statistical analysis was conducted using R software (version 4.2.2, https://www.r-project.org/). For continuous variables, the Shapiro-Wilk test was used for assessing distribution. If the data followed a normal distribution, parametric tests were applied; otherwise, non-parametric tests were used. Categorical variables were analyzed using the chi-square test or Fisher’s exact test (where applicable). Categorical and continuous variables were presented as frequencies (percentages), mean ± standard deviation, or median (interquartile range) as appropriate. Gradient-weighted Class Activation Mapping (Grad-CAM) is utilized for visualizing and understanding the decision-making process within DL. The SHapley Additive exPlanations (SHAP) method is employed to explain the influence and contribution of features on the fused model’s output. The construction, training, and validation of the deep convolutional neural network model were carried out using the PyTorch framework (version 1.13.0, https://pytorch.org/).

### Evaluation methods

**Figure 2:**
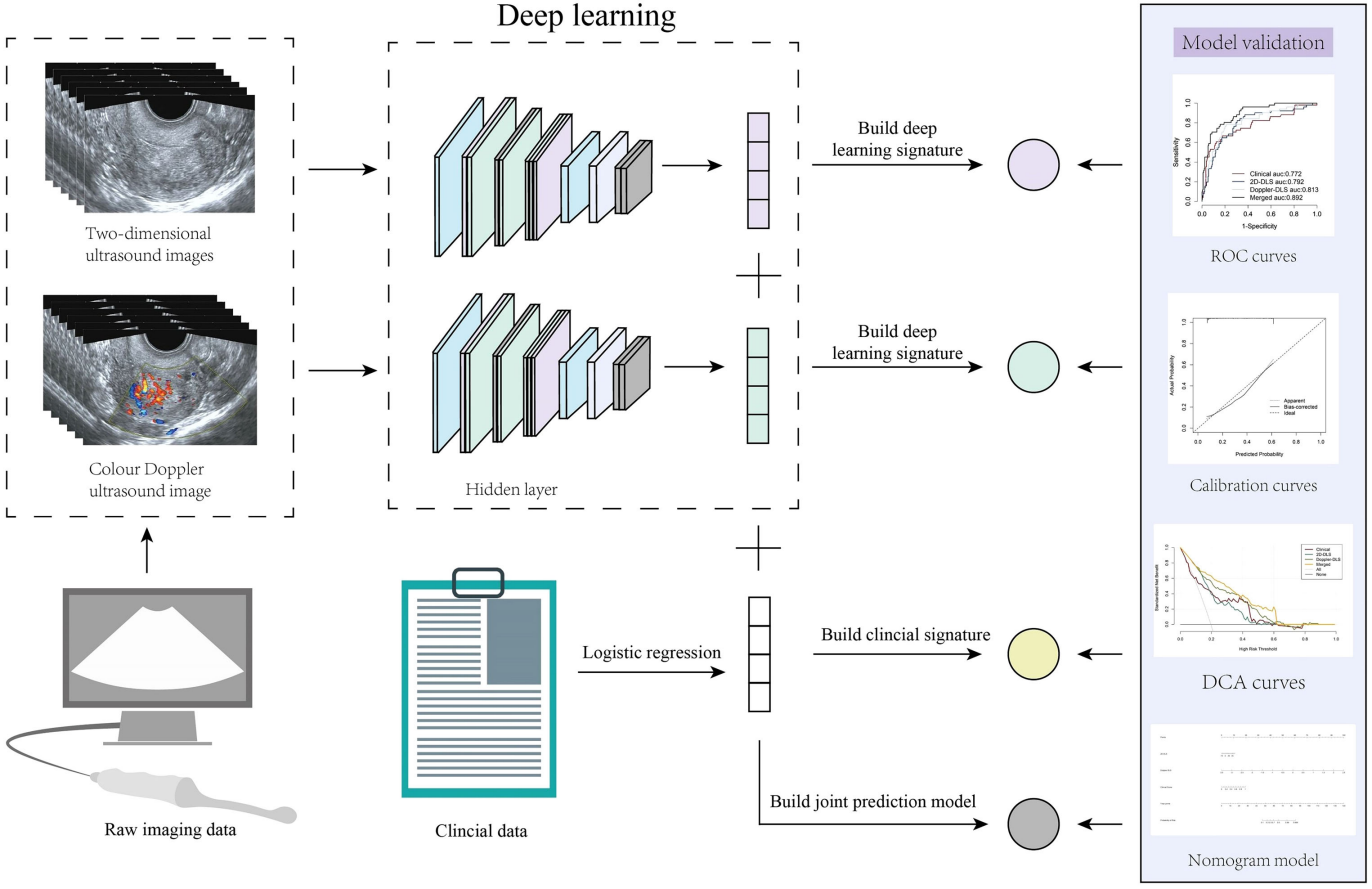
Overall study workflow. This figure illustrates the work analysis flow of the entire study. Endometrial cancer prediction was achieved by integrating transvaginal ultrasound images with clinical data using deep learning (DL) techniques. Initially, two types of ultrasound images, two-dimensional grayscale and color Doppler, were processed through a DL model to generate DL signatures. Simultaneously, clinical data were analyzed using logistic regression to create a clinical signature. These signatures were subsequently combined to construct a merged prediction model. The model’s performance was validated using the area under the receiver operating characteristic curve (AUC), calibration curves, and decision curve analysis, and the resulting predictive model was visualized as a nomogram.

The diagnostic models were evaluated using a range of performance metrics, including the area under the AUC with its 95% confidence interval, accuracy, sensitivity, specificity, and F1 score. The Delong test was employed to compare the performance of different models and assess statistical significance. Model fitness was determined using the Hosmer-Lemeshow goodness-of-fit test and calibration curves. Additionally, decision curve analysis (DCA) was performed to evaluate the net clinical benefit of the predictive models. A nomogram was constructed to visualize the combined model. Furthermore, several ultrasound specialists were involved in assessing the clinical applicability and interpretability of the combined model in predicting EC. **Figure 2** illustrates the workflow of the entire study.

## Results

**Figure 3:**
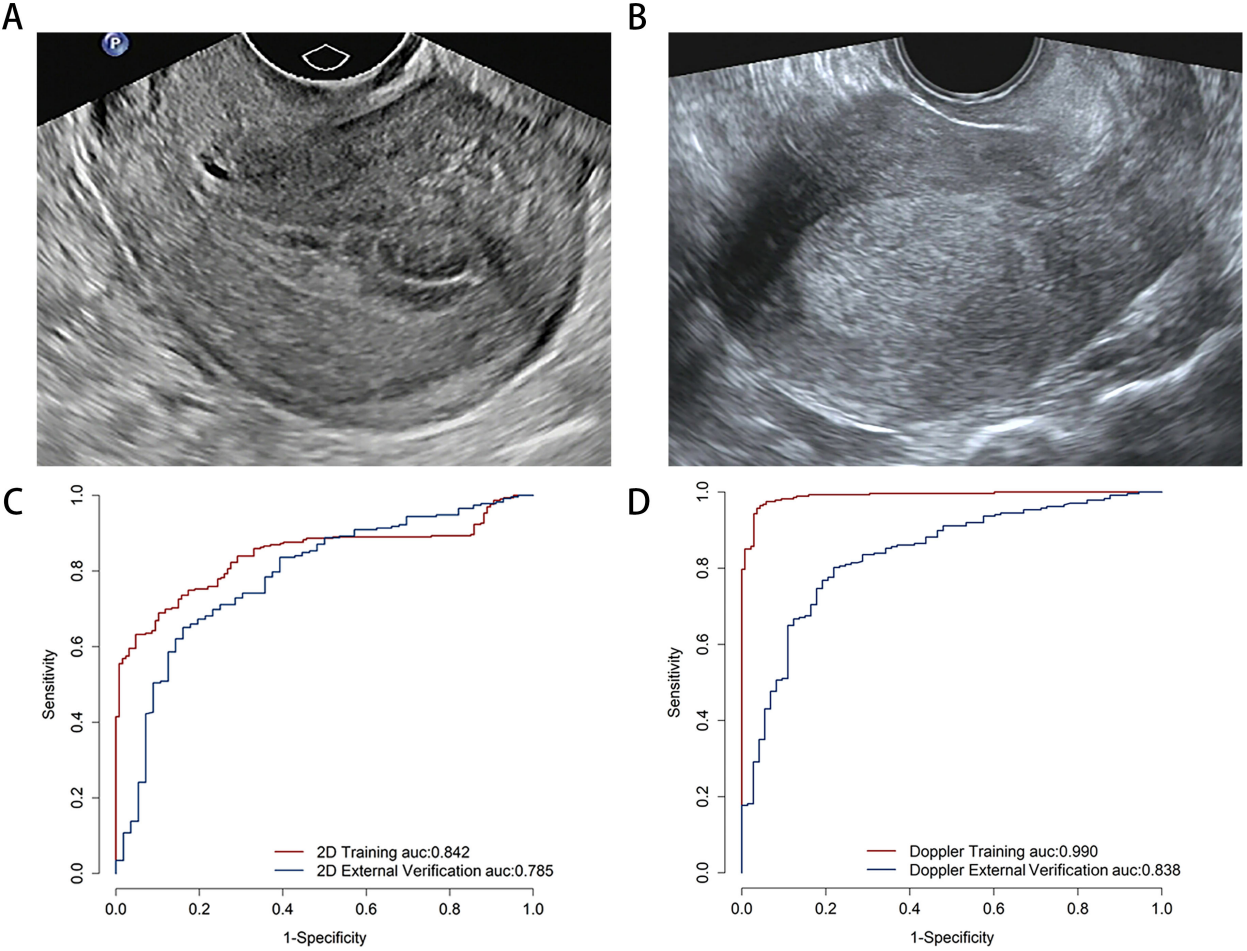
The examples of transvaginal ultrasound images for non-endometrial cancer **(A)** and endometrial cancer **(B)**. Comparison predictive performance for image-level deep learning model. The area under the receiver operating characteristic curve for two-dimensional ultrasound **(C)** and color Doppler **(D)** models on the image-level.

This study included 1,443 images obtained from 610 cases across two centers. The training set comprised 351 cases (81 cases of EC and 270 cases of non-EC), while the external validation set consisted of 260 cases (51 cases of EC and 209 cases of non-EC). Baseline data, including age, body mass index (BMI), gravidity, fertility, menopausal status, irregular vaginal bleeding, hypertension, diabetes, hypothyroidism, and polycystic ovary syndrome (PCOS) are presented in **Table 1**. Examples of TVU images are presented in **Figures 3A, B**.

**Table 1:** Baseline data.

Variables	Training set (351)		External validation set (260)	
	EC (81)	nEC (270)	EC (51)	nEC (209)
Age	51.94 ± 11.12	42.94 ± 9.33	60.04 ± 9.95	45.63 ± 12.89
BMI	25.52 ± 4.79	23.22 ± 3.70	25.75 ± 3.76	23.45 ± 3.57
gravidity				
No	11 (13.6%)	29 (10.7%)	3 (5.9%)	59 (28.2%)
Yes	70 (86.4%)	241 (89.3%)	48 (94.1%)	150 (71.8%)
Fertility				
No	13 (16.0%)	43 (15.9%)	9 (17.6%)	63 (30.1%)
Yes	68 (84.0%)	227 (84.1%)	42 (82.4%)	146 (69.9%)
Menopausestatus				
No	36 (44.4%)	237 (87.8%)	14 (27.5%)	154 (73.7%)
Yes	45 (55.6%)	34 (12.2%)	37 (72.5%)	55 (26.3%)
Irregular vaginal bleeding				
No	16 (19.8%)	168 (62.2%)	2 (3.9%)	101 (48.3%)
Yes	65 (80.2%)	102 (37.8%)	49 (96.1%)	108 (51.7%)
Hypertension				
No	55 (67.9%)	244 (90.4%)	26 (51.0%)	174 (83.3%)
Yes	26 (32.1%)	26 (9.6%)	25 (49.0%)	35 (16.7%)
Diabetes				
No	64 (70.0%)	263 (97.4%)	35 (68.6%)	198 (94.7%)
Yes	17 (21.0%)	2.6 (29.2%)	16 (31.4%)	11 (5.3%)
Hypothyroidism				
No	80 (98.8%)	266 (98.5%)	49 (96.1%)	204 (97.6%)
Yes	1 (1.2%)	4 (1.5%)	2 (3.9%)	5 (2.4%)
PCOS				
No	79 (97.5%)	266 (98.5%)	51 (100%)	208 (99.5%)
Yes	2 (2.5%)	4 (1.5%)	0 (0)	1 (0.5)
FIGO Stage (2009)				
I	61 (75.3%)	–	37 (72.5%)	–
II	6 (7.4%)	–	4 (7.8%)	–
III	12 (14.8%)	–	9 (17.6%)	–
IV	2 (2.5%)	–	1 (2.1%)	–

Data were presented as No. (%) and mean ± SD.

### Clinical model construction

Univariate and multivariate regression analyses were performed to identify statistically significant associations between various variables and the presence of EC. Odds ratios (OR) and corresponding p-values were calculated to assess the effects of these variables. Specifically, age, BMI, menopausal status, irregular vaginal bleeding, hypertension, and diabetes were statistically significant in the univariate analysis. In the multivariate analysis, BMI, menopausal status, irregular vaginal bleeding, and diabetes remained significant. These variables were subsequently selected as optimal features for constructing the clinical model (**Table 2**).

**Table 2:** Results of univariate and multivariate regression analysis of clinical information.

Variables	Univariate logistic regression		Multivariate logistic regression	
	OR(CI)	P Value	OR(CI)	P Value
Age	1.09(1.06,1.12)	<0.001	1.03(0.99,1.07)	0.156
BMI	1.15(1.08,1.22)	<0.001	1.10(1.02,1.19)	0.011
Gravidity	0.77(0.36,1.61)	0.482		
Fertility	0.99(0.50,1.95)	0.979		
Menopause status	8.98(5.08,15.87)	<0.001	3.71(1.59,8.61)	0.002
Irregular Vaginal bleeding	6.69(3.67,12.19)	<0.001	4.93(2.53,9.60)	<0.001
Hypertension	4.44(2.39,8.22)	<0.001	1.41(0.64,3.11)	0.397
Diabetes	9.98(3.97,25.08)	<0.001	3.97(1.18,13.30)	0.026
Hypothyroidism	0.83(0.09,7.54)	0.870		
PCOS	1.68(0.30,9.36)	0.552		

PCOS, polycystic ovary syndrome.

### The performance of image-level deep learning

The predictive performance of the DL model was evaluated at the image-level using multimodal ultrasound. On the training set, the 2D ultrasound model (2D-DLS) achieved an area under the curve (AUC) of 0.842 (95% CI, 0.805–0.879), with an accuracy of 0.751, a sensitivity of 0.689, and a specificity of 0.898. In contrast, the color Doppler (Doppler-DLS) model yielded an AUC of 0.990 (95% CI, 0.983–0.997), an accuracy of 0.959, a sensitivity of 0.957, and a specificity of 0.964. On the external validation set, the 2D ultrasound model showed an AUC of 0.785 (95% CI, 0.718–0.853), an accuracy of 0.688, a sensitivity of 0.651, and a specificity of 0.839, while the color Doppler model obtained an AUC of 0.838 (95% CI, 0.786–0.889), an accuracy of 0.797, a sensitivity of 0.802, and a specificity of 0.781 (**Figures 3C, D**).

### Patient-level model performance on training and external validation sets

**Figure 4:**
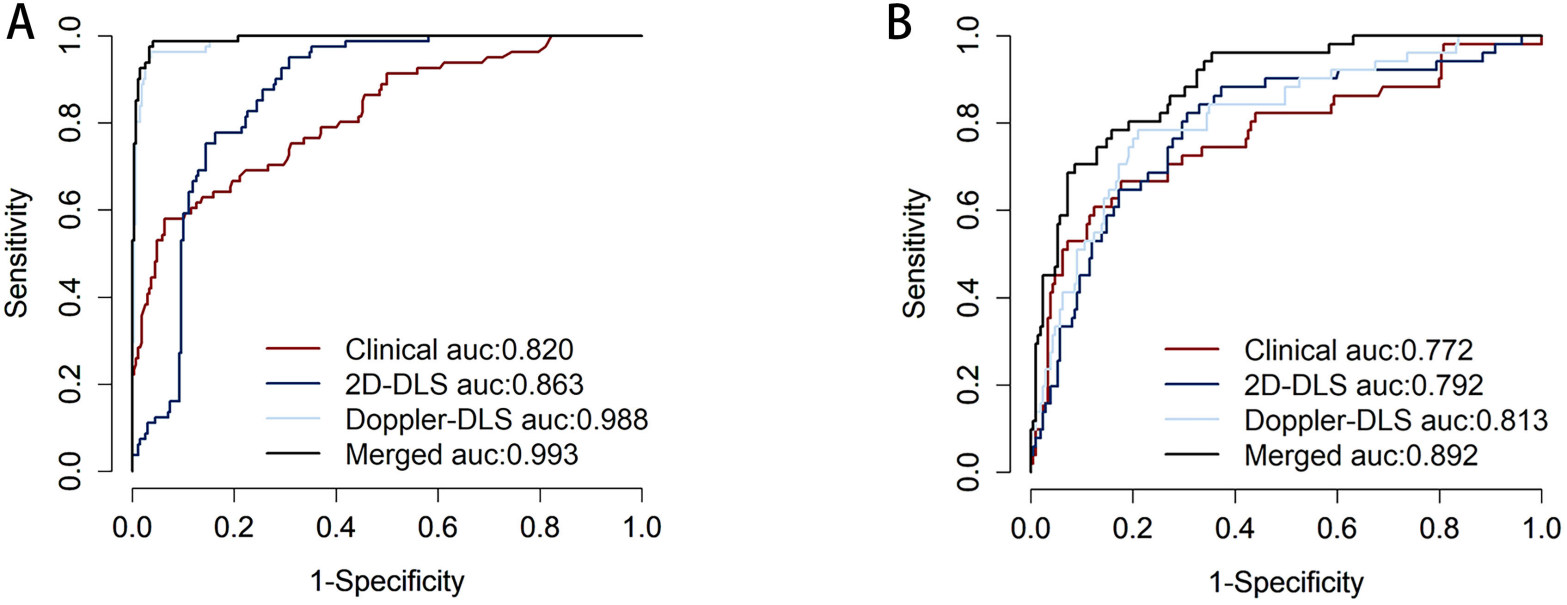
Displays the receiver operating characteristic (ROC) curve performance of different models on the training set **(A)**, where the clinical model (red) has an area under the ROC curve (AUC) of 0.820, indicating good predictive ability but falling short compared to other models. The 2D-DLS model (blue) shows improved performance with an AUC of 0.863. The Doppler-DLS model (light blue) exhibits significantly superior performance, achieving an AUC of 0.988. The Merged model (black) reaches an AUC of 0.993, nearly approaching perfect classification, indicating it has the strongest predictive capability in the training set. Corresponds to the ROC curve for the external validation set **(B)**. Where the clinical model (red) has an AUC of 0.772, indicating good predictive ability but falling short compared to other models. The 2D-DLS model (blue) shows improved performance with an AUC of 0.792. The Doppler-DLS model (light blue) exhibits significantly superior performance, achieving an AUC of 0.813. The Merged model (black) reaches an AUC of 0.892.

The performance of the patient-level models on the training set and external validation set was as follows: On the training set, the clinical model achieved an AUC of 0.820 (0.765, 0.874) with sensitivity of 0.580 and specificity of 0.937, the 2D ultrasound model had an AUC of 0.863 (0.825, 0.902) with sensitivity of 0.951 and specificity of 0.693, and the color doppler model showed an AUC of 0.988 (0.978, 0.997) with sensitivity of 0.963 and specificity of 0.963. The combined model performed the best with an AUC of 0.993 (0.986, 0.999), sensitivity of 0.988, and specificity of 0.959. On the external validation set, the clinical model had an AUC of 0.772 (0.690, 0.854) with sensitivity of 0.667 and specificity of 0.823, the 2D ultrasound model achieved an AUC of 0.792 (0.719, 0.864) with sensitivity of 0.824 and specificity of 0.694, and the color doppler model showed an AUC of 0.813 (0.745, 0.881) with sensitivity of 0.784 and specificity of 0.789. The merged model exhibited superior performance, achieving an AUC of 0.892 (95% CI: 0.846–0.938), a sensitivity of 0.784, and a specificity of 0.842. It outperformed the individual models across all evaluated metrics, demonstrating the effectiveness of integrating clinical data with multimodal ultrasound imaging (**Table 3**; **Figure 4**). To assess the robustness of the model, we conducted subgroup analyses stratified by menopausal status, age groups (>50 years vs. ≤50 years), and BMI categories (>24 kg/m² vs. ≤24 kg/m²). Detailed results are presented in **Supplementary Figure S2**.

**Table 3:** Performance results on the training set and external validation set.

Model	Training set					External validation set				
	AUC(CI)	ACC	SEN	SPE	F1	AUC(CI)	ACC	Sen	Spe	F1
Clinical	0.820 (0.765, 0.874)	0.855	0.58	0.937	0.648	0.772 (0.690, 0.854)	0.792	0.667	0.823	0.557
2D-DLS	0.863 (0.825, 0.902)	0.752	0.951	0.693	0.639	0.792 (0.719, 0.864)	0.719	0.824	0.694	0.535
Doppler-DLS	0.988 (0.978, 0.997)	0.963	0.963	0.963	0.923	0.813 (0.745, 0.881)	0.788	0.784	0.789	0.593
Merged	0.993 (0.986, 0.999)	0.966	0.988	0.959	0.93	0.892 (0.846, 0.938)	0.831	0.784	0.842	0.645

AUC, the area under the receiver operating characteristic curve; Acc, accuracy, Acc = True Positives + True Negatives)/(True Positives + True Negatives + False Positives + False Negatives); Sen, sensitivity, Sen = True Positives/(True Positives+ False Negatives); Spe, specificity, Spe = True Negatives/(True Negatives + False Positives); F1 Score = 2 * (Precision * Recall)/(Precision + Recall); CI, confidence interval.

### Model fitting verification

**Figure 5:**
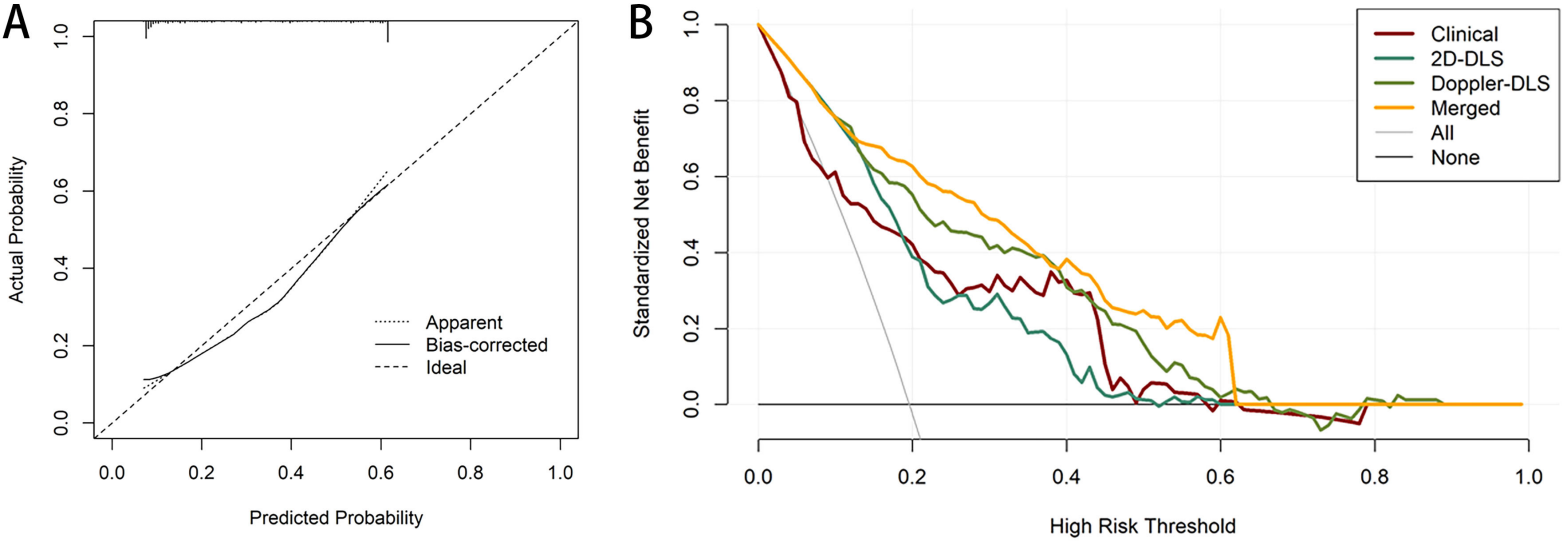
Calibration and decision curve analysis (DCA) for the merged model. The calibration curves for the merged model which indicate the goodness-of-fit of the model **(A)**. DCA for four models predicting endometrial cancer **(B)**.

Calibration curves were employed to assess the agreement between the predicted and actual outcomes, and the merged model demonstrated excellent calibration ability. The predicted probabilities closely aligned with the actual occurrence probabilities, approaching an ideal calibration, which indicates that the model’s predictions are both reliable and generalizable (**Figure 5A**). We explored the merged model’s predictive capability in distinguishing between non-EC and different cancer stages. Specifically, we conducted separate binary classification analyses to calculate the AUC for the merged model in distinguishing non-EC from each of the subsequent stages (1-4). The results indicate good performance, showing an AUC range of 0.87–0.97 (**Supplementary Figure S3**).

**Figure 6:**
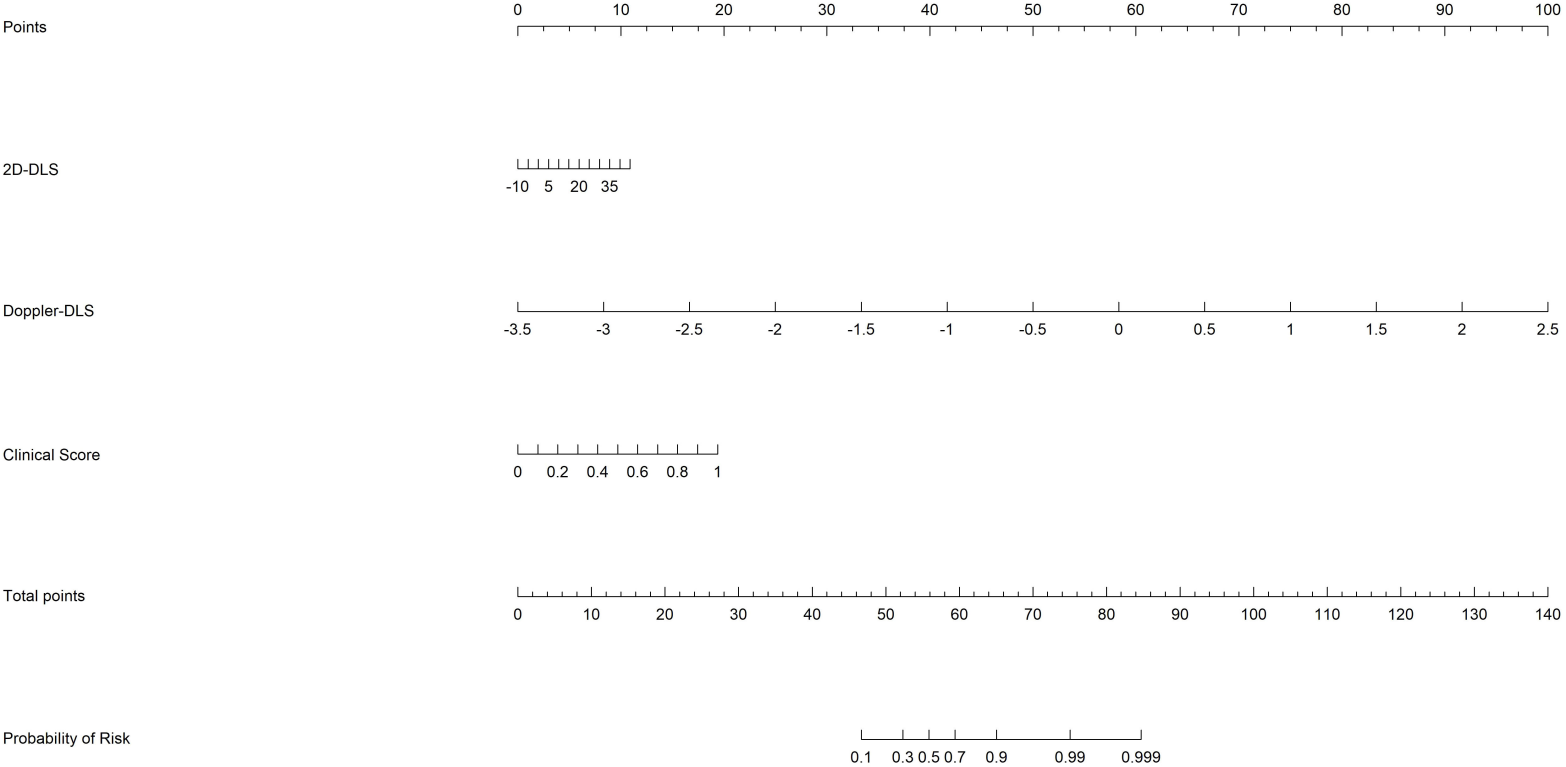
The nomogram based on risk scores of the from the clinical, 2D ultrasound, and Doppler ultrasound models.

Decision curve analysis (DCA) further demonstrated the clinical utility of the models. The DCA showed that the net benefit of the clinical model (red line) decreased as the high-risk threshold increased, although it remained relatively stable across most of the range. The 2D DL system (green line) provided a higher net benefit than the clinical model within the moderate-risk threshold range (0.2–0.4), despite a slight performance decline at higher thresholds. The Doppler DL system (yellow line) performed well in the low-risk threshold range; however, its net benefit diminished at higher thresholds, eventually falling behind the merged model. The merged model (orange line) outperformed the other models, particularly in the low-to-moderate risk thresholds, by providing a higher standardized net benefit and demonstrating more substantial decision-making advantages across various thresholds. The “All” (gray) and “None” (black) lines represent the decision baselines for scenarios in which either all patients are considered high-risk or none are. Overall, the merged model provided the highest net benefit across most thresholds, underscoring its strong clinical utility for risk prediction in this population (**Figure 5B**). Finally, a nomogram was constructed based on the risk scores derived from the three models to facilitate visual assessment by clinicians (**Figure 6**).

**Figure 7:**
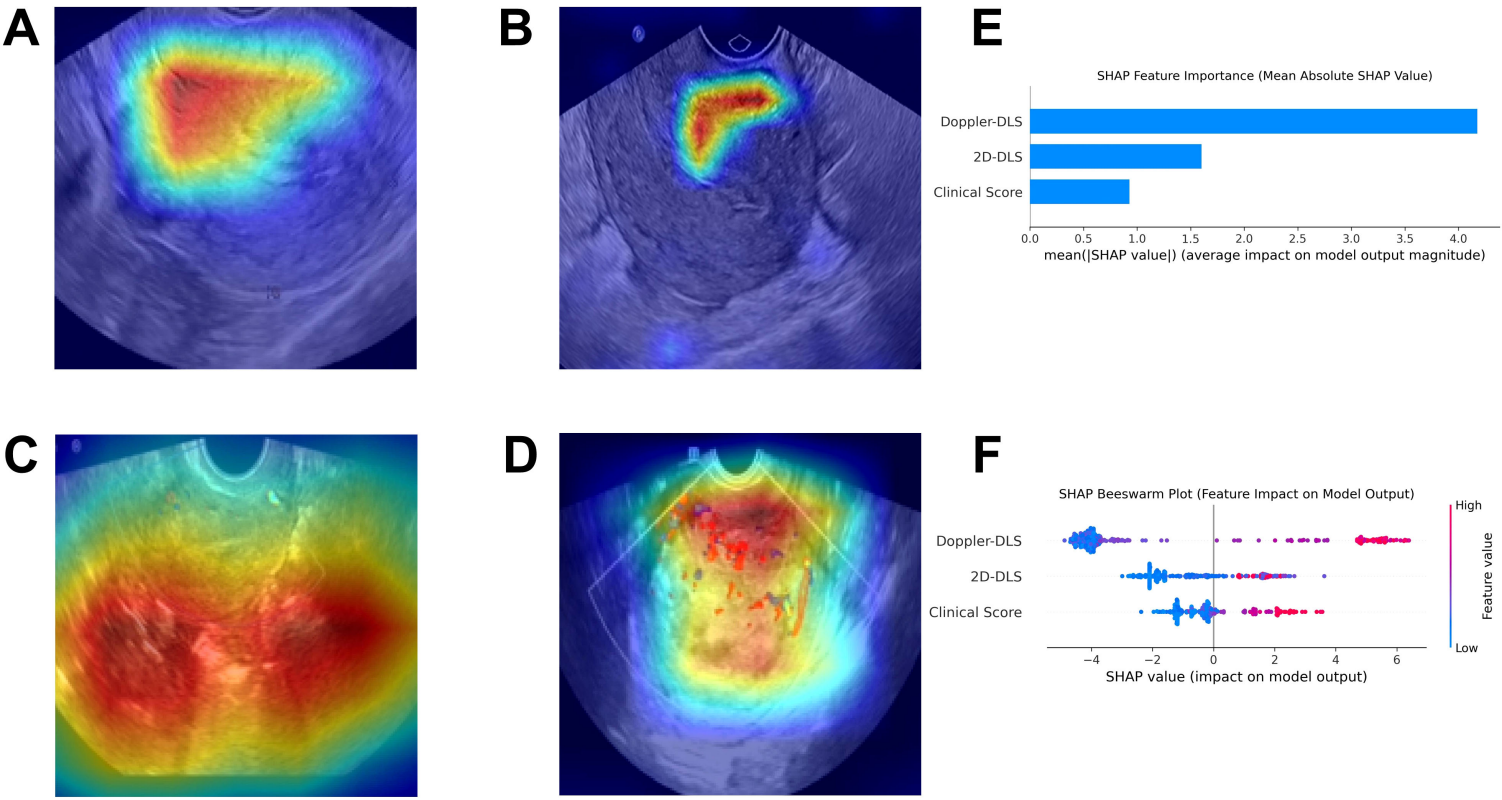
The explainable AI plots. Gradient-weighted Class Activation Mapping visualizes the model’s attention zones as heatmaps for non-endometrial cancer **(A)** and endometrial cancer **(B)** in 2D ultrasound, and for non-endometrial cancer **(C)** and endometrial cancer **(D)** in color Doppler ultrasound **(A–D)**. The SHapley Additive exPlanations method identified the key feature contributions to the merged model, ranking Doppler-DLS, 2D-DLS, and Clinical Score as the top three most important **(E)**. The Beeswarm plot **(F)** further illustrated that higher feature values positively influenced the model’s output.

Grad-CAM visualized the DL model’s decision-making process as heatmaps, where hot areas indicated the model’s attention regions (**Figures 7A-D**). SHAP explained the ranked contributions of features to the fused model (**Figure 7E**) and relationship among their own feature value impact on the model’s output (**Figure 7F**).

## Discussion

EC is recognized as the most prevalent malignancy of the female reproductive system, imposing significant health risks and considerable socioeconomic burdens (21). The disease is particularly concerning due to its insidious nature and frequent diagnosis at advanced stages, which complicates treatment and adversely affects prognosis. In light of these challenges, we employed a multicenter, retrospective approach using advanced DL techniques to analyze ultrasound images in combination with clinical data, with the goal of constructing a robust predictive model for early disease identification.

In this study, we demonstrate the feasibility of using DL techniques to analyze ultrasound images and predict EC at both the image and patient levels. At the image level, the AUC of the 2D ultrasound DL model on the external validation set reached 0.785 (95% CI, 0.718–0.853), while the color Doppler DL model performed better with an AUC of 0.838 (95% CI, 0.786–0.889). Similarly, at the patient level, the 2D ultrasound DL model achieved an AUC of 0.792 (95% CI, 0.719–0.864), and the color Doppler DL model reached an AUC of 0.813 (95% CI, 0.745–0.881). Notably, the models based on DL analysis outperformed those based solely on clinical data. More importantly, integrating multimodal data markedly enhanced model performance, with the fusion model achieving an AUC of 0.892 (95% CI, 0.846–0.938). The Delong test confirmed that the AUC of the fusion model was significantly superior to those of the single-mode models (p < 0.05). These findings indicate that this multifaceted approach not only improves diagnostic precision but also lays the groundwork for personalized patient management strategies, ultimately leading to better clinical outcomes for those at risk of developing EC (22).

Our findings reveal significant differences in the complex, often subtle, sonographic imaging patterns captured by CNNs between patients with EC and those without. This underscores the utility of ultrasound imaging combined with advanced analytical techniques, such as radiomics, as a non-invasive diagnostic tool for clinicians. Previous research has highlighted that specific ultrasound features, such as vascular patterns and tissue texture, are closely associated with malignant transformations in gynecological cancers, including endometrial carcinoma (23). By integrating these imaging biomarkers with clinical data, our approach significantly enhances the capabilities for early diagnosis, which may lead to better patient outcomes through timely interventions and management strategies (24). Moreover, various DL architectures (e.g., ResNet and EfficientNet) have been shown to influence predictive performance, with some architectures demonstrating a superior ability to generalize from training data to unseen validation datasets (25). These results align with previous studies where DL approaches have been successfully applied to diverse medical imaging tasks, highlighting the transformative potential of artificial intelligence in oncology diagnostics (26–28).

Additionally, the identification of clinical risk factors—such as BMI, menopausal status, and irregular vaginal bleeding—as significant predictors of EC adds another important dimension to our predictive framework (29, 30). These findings are consistent with existing literature that has reported similar associations between these factors and an increased risk of cancer (31). A deeper understanding of the interplay between these clinical risk factors and imaging characteristics can further refine predictive models while enhancing our knowledge of EC epidemiology. Future studies should explore the biological mechanisms underlying these associations, as this could lead to the discovery of novel preventive strategies or therapeutic targets for high-risk populations (32).

Distinguishing EC from benign conditions in ultrasound imaging is challenging, as the manifestations on 2D and Doppler imaging can be overlapping. The integration of DL technology offers a promising solution by addressing the critical need for improved diagnostic methods amidst the rising incidence of EC. Building upon previous AI research in gynecologic cancers, our study advances this field by utilizing a larger dataset and more sophisticated DL models, thereby enhancing the robustness of our findings compared to earlier studies. This collaborative approach is imperative for advancing the field of medical imaging and improving outcomes for patients with EC and other malignancies (33–35).

This study presents several limitations that warrant consideration. First, the participant pool was derived from only two medical centers, and class imbalance was observed in this study (with only 132 EC cases). These factors may limit the generalizability of our findings to broader populations. Second, the study centers were from the same region (Guangzhou, China), which might limit the generalizability of our findings to other populations or healthcare systems with different ethnic profiles, lifestyles, or ultrasound practices. Furthermore, the absence of long-term follow-up data presents challenges in assessing the sustained predictive validity of the developed models. Therefore, further validation in larger, more diverse, and multi-ethnic cohorts is necessary to enhance both the robustness and clinical relevance of our findings (36).

In conclusion, our research integrates DL-based ultrasound imaging features with clinical risk factors to develop a novel predictive model for the early diagnosis of EC. The observed improvement in predictive accuracy underscores the potential of this model to significantly aid in clinical decision-making and patient management. Future studies should focus on larger, multicenter validations to confirm the model’s applicability across varied populations and clinical settings, thereby facilitating its integration into routine clinical practice for enhanced patient outcomes.

## Data Availability

The original contributions presented in the study are included in the article/**Supplementary Material**. Further inquiries can be directed to the corresponding authors.
