# Supplementary material for: Integrating deep learning and clinical characteristics for early prediction of endometrial cancer using multimodal ultrasound imaging: a multicenter study
Source: Front Oncol. 2025 Jul 8;15:1600242. doi: 10.3389/fonc.2025.1600242 (PMC12279480; doi:10.3389/fonc.2025.1600242)
Supplement: Supplementary file 1 [file DataSheet1.docx]

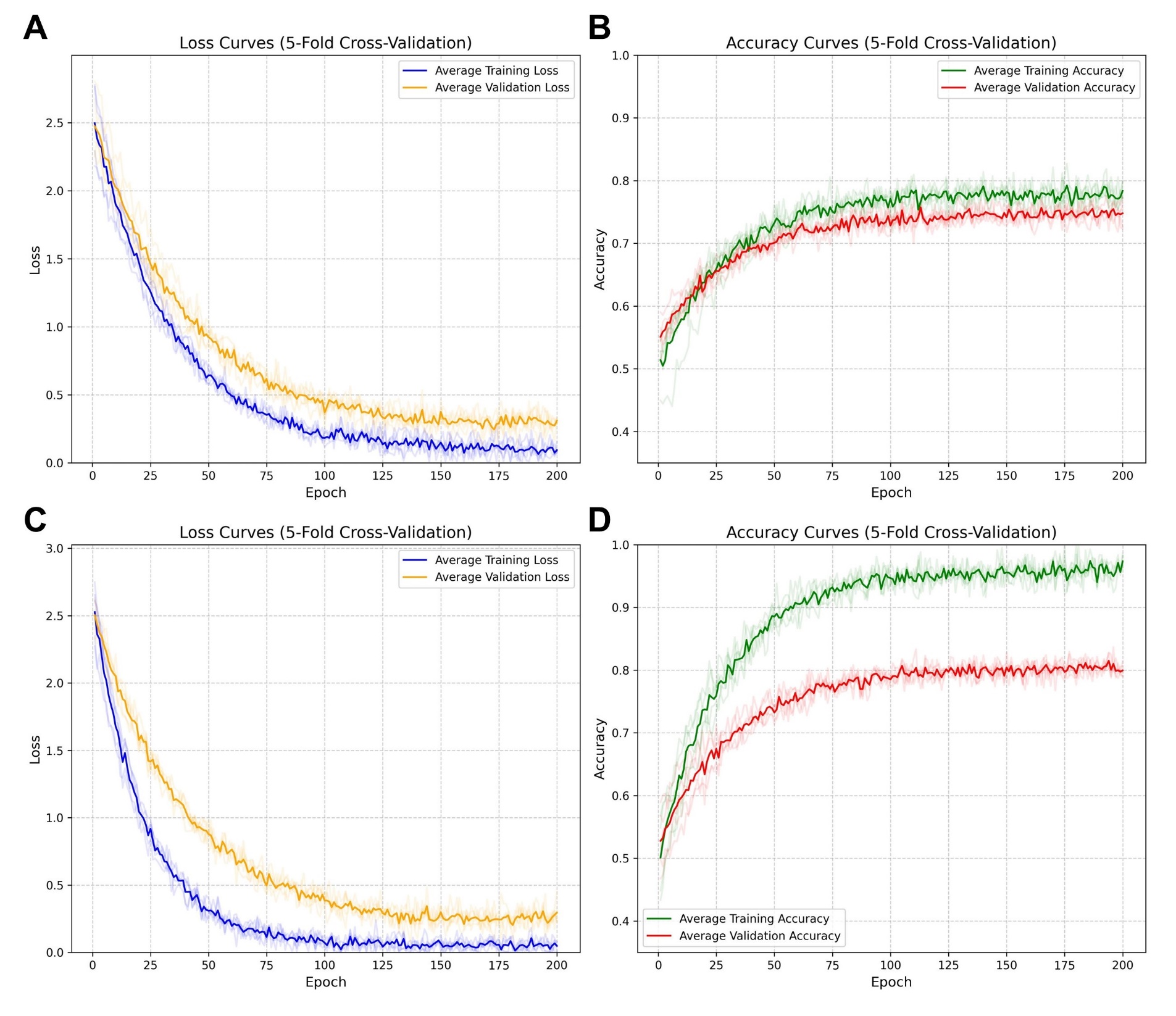


**Figure S1. Deep learning model training and validation curves.** Average training and validation loss and accuracy curves for the deep learning (DL) model during the 5-fold cross-validation strategy. Average training and validation loss (A) and accuracy (B) curves for the optimal DL model in the two-dimensional ultrasound modality. Average training and validation loss (C) and accuracy (D) curves for the optimal DL model in the color Doppler ultrasound modality.


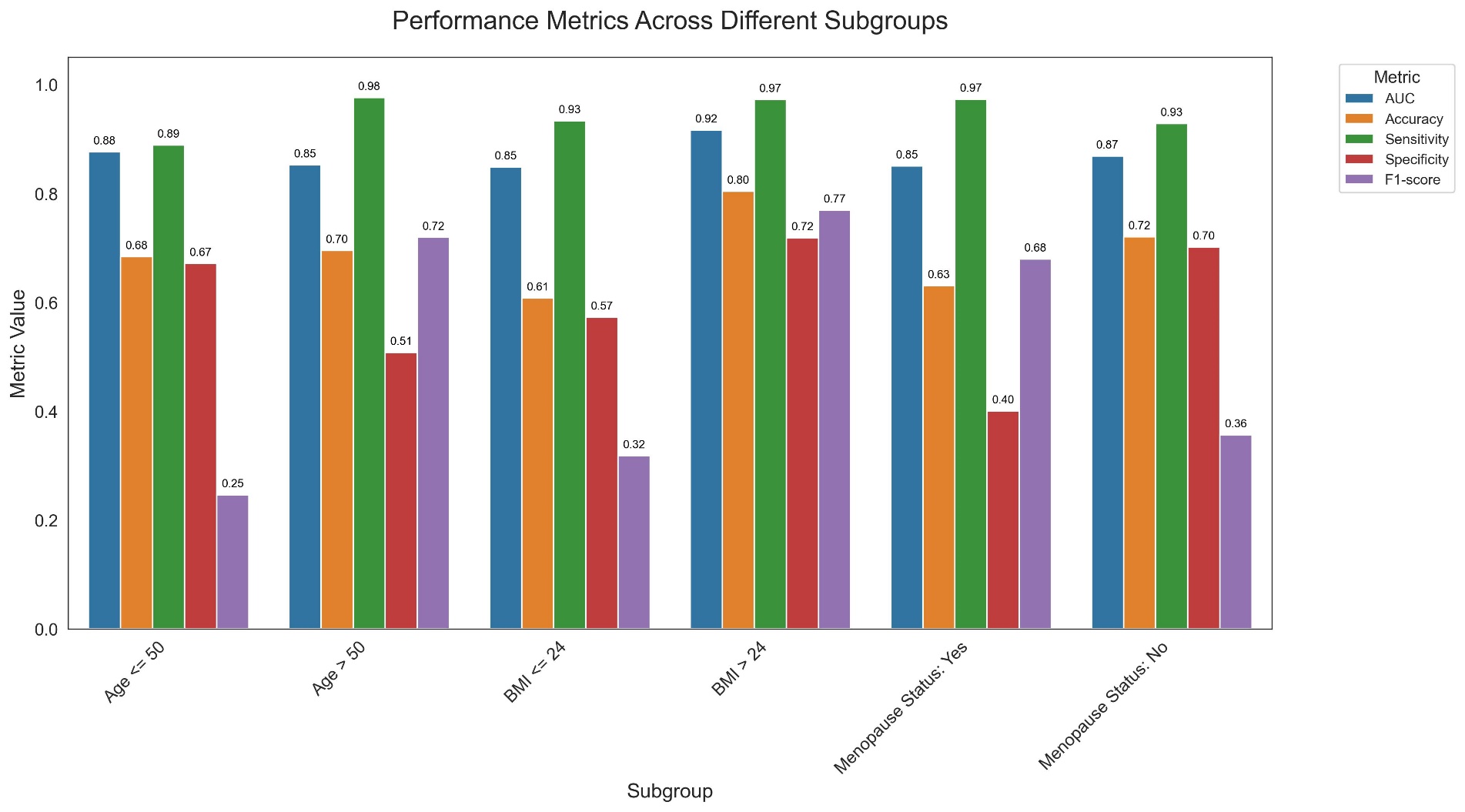


**Figure S2.** Bar chart of fusion model performance in subgroup analyses.


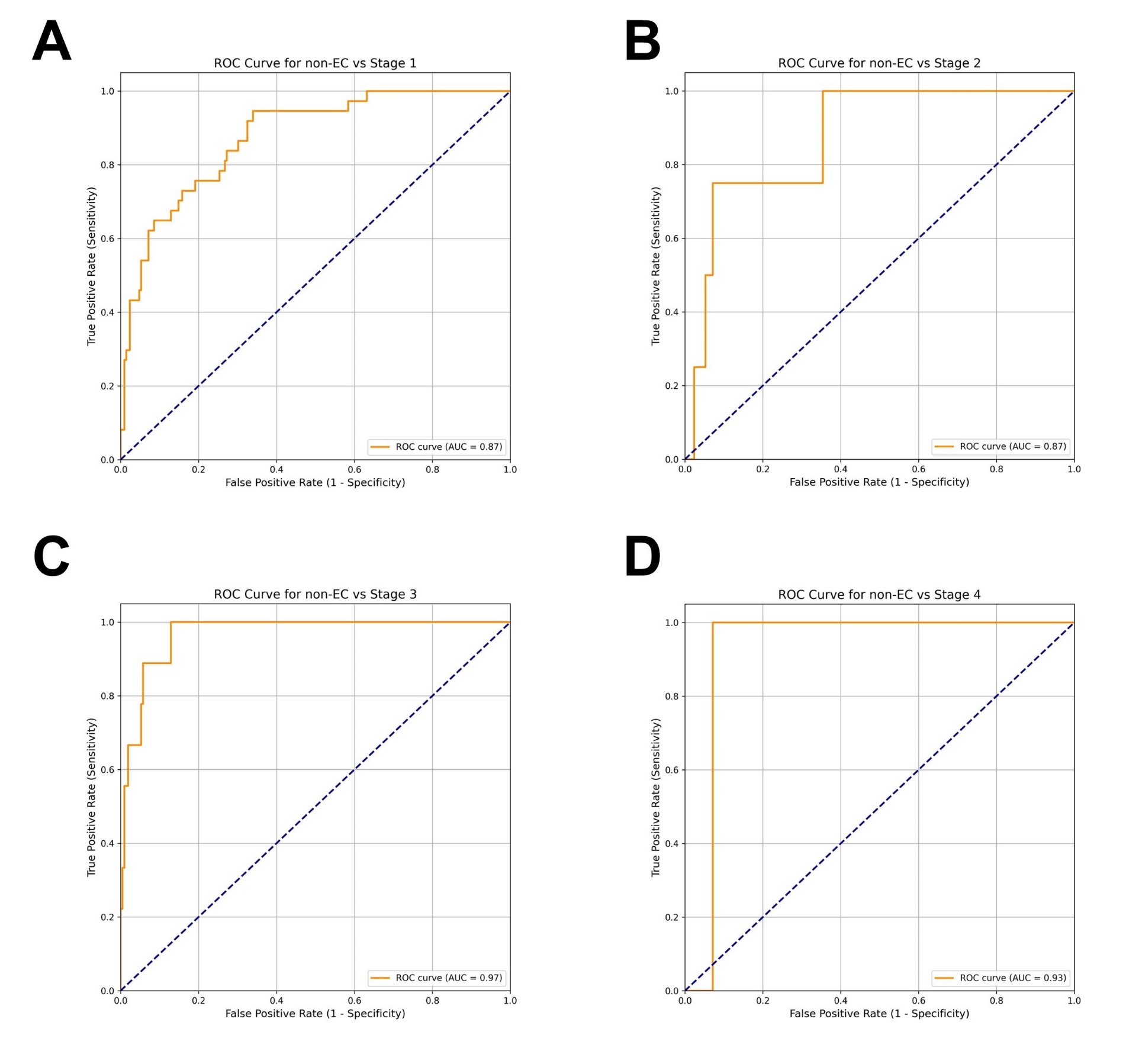


**Figure S3.** To validate the merged model's predictive performance across pathological staging subgroups, the area under the receiver operating characteristic curve (AUC) was calculated for distinguishing non-endometrial cancer from Stage 1 (AUC = 0.87, A), Stage 2 (AUC = 0.87, B), Stage 3 (AUC = 0.97, C), and Stage 4 (AUC = 0.93, D).
